# The role of METTL3 in transposable elements regulation and 2C-like program induction in mouse embryonic stem cell

**DOI:** 10.1186/s13619-025-00262-w

**Published:** 2025-11-20

**Authors:** Xiuyu Chen, Bingqiu Chen, Yingying Zhao, Ziyi Wen, Jiajie Hao, Lingmei Jin, Danfeng Li, Xiongzhi Quan, Kaixin Wu, Mingqiang Deng, Xichen Bao, Jie Wang, Jiekai Chen

**Affiliations:** 1 Center for Biomedical Digital Science, Guangdong Provincial Key Laboratory of Stem Cell and Regenerative Medicine, Guangdong-Hong Kong Joint Laboratory for Stem Cell and Regenerative Medicine, Guangzhou Institutes of Biomedicine and Health, Chinese Academy of Sciences, Guangzhou, 510530 China; 2 University of Chinese Academy of Sciences, Beijing, 100049 China; 3 Centre for Regenerative Medicine and Health, Hong Kong Institute of Science and Innovation, Chinese Academy of Sciences, Hong Kong SAR, 999077 China; 4 Joint School of Life Sciences, Guangzhou Institutes of Biomedicine and Health, Chinese Academy of Sciences, Guangzhou 510530, China, Guangzhou Medical University, Guangzhou, 511436 China; 5 The Fifth Affiliated Hospital of Guangzhou Medical University, Guangzhou, China; 6 Center for Cell Lineage Atlas, Guangdong Provincial Key Laboratory of Stem Cell and Regenerative Medicine, Guangdong-Hong Kong Joint Laboratory for Stem Cell and Regenerative Medicine, GIBH-CUHK Joint Research Laboratory On Stem Cell and Regenerative Medicine, State Key Laboratory of Respiratory Disease, Guangzhou Institutes of Biomedicine and Health, Chinese Academy of Sciences, Guangzhou, 510530 China; 7 School of Life Sciences, Precise Genome Engineering Center, Guangzhou University, Guangzhou, China

**Keywords:** METTL3, M^6^A modification, Transposable elements, 2C-like state

## Abstract

METTL3 is a crucial mRNA methyltransferase in mammals, essential for the regulation of gene expression and various biological processes. Here, we demonstrate that *Mettl3* knockout (KO) in mouse embryonic stem cells (mESCs) leads to widespread upregulation of transposable elements (TEs) and 2-cell (2C)-like genes in a m^6^A enzyme activity-dependent manner, independent of culture conditions. Furthermore, embryo chimera experiments using a transient METTL3 degradation system (dTAG) revealed that METTL3-deficient mESCs can contribute to trophectoderm lineages at the blastocyst stage, indicative of expanded developmental potential. These findings highlight the role of METTL3-mediated m^6^A modifications in regulating the transcriptional and developmental plasticity of mESCs and suggest a link between m^6^A loss and the acquisition of a 2C-like state with features of extended potency.

**Supplementary Information:**

The online version contains supplementary material available at 10.1186/s13619-025-00262-w.

## Background

N6-methyladenosine (m^6^A) is a prevalent modification found in messenger RNA (mRNA) in mammals, plants, and other eukaryotes (Jia et al. 2013). This modification plays a critical role in regulating gene expression and biological processes. METTL3 is the main component responsible for enzymatic catalytic activity in the m^6^A methyltransferase complex (Wang et al. 2016; Mansfield 2024). The METTL3 protein in eukaryotes is highly conserved, particularly the catalytic active site DPPW (Wang et al. 2016; Huang and Yin 2018; Akhtar et al. 2021), mutations or knockout of which can lead to a nearly complete loss of the ability of METTL3 to catalyze m^6^A modification (Wang et al. 2016).

Transposable elements (TEs) are a class of DNA sequences that can change their positions within the genome, accounting for approximately 45% of the genome sequence in both humans and mice. TEs include several major families, such as LINEs, SINEs and endogenous retroviral elements (ERVs) (Cordaux and Batzer 2009; Hutchins and Pei 2015). TE-derived RNAs are highly enriched with m^6^A modifications (Liu et al. 2020). In mouse embryonic stem cells (mESCs), the knockout of the RNA m^6^A methyltransferase *Mettl3* leads to transcriptional activation of TEs (Liu et al. 2021; Xu et al. 2021). The m^6^A modification on TE RNAs can recruit the H3K9 methyltransferase SETDB1 through its reader protein YTHDC1, facilitating the deposition of the histone modification H3K9me3 at these loci, thereby repressing TE RNA transcription (Liu et al. 2021). Knockout of *Ythdc1* results upregulation of TEs, including LINEs and most H3K9me3 deposited ERVs, such as IAP and MMERVK10c families (Liu et al. 2021; Chen et al. 2021). This highlights a mechanism of heterochromatin regulation in mammals.

However, one study reported that *Mettl3* knockout in mESCs resulted in the upregulation of IAP mRNA levels, while transcripts of LINEs exhibited a decreasing tendency (Chelmicki et al. 2021). Similarly, another study demonstrated that *Mettl3* knockout in mESCs downregulates LINE RNA levels, particularly L1 elements (Xu et al. 2021). There are many issues, such as knockout strategy, culture condition and mESCs heterogeneity, which can lead to the varieties among labs. However, as demonstrated in our previous studies, rigorous validation of the cell lines is essential for reliably reproducing LINE1 activation following *Mettl3* knockout, including confirming the knockout strategy by measuring m⁶A levels via mass spectrometry and validating the maintenance of mESC pluripotency through embryonic injection (Wu et al. 2020; Liu et al. 2021; Xi et al. 2025). Further experimental evidence is required to elucidate the molecular mechanisms by which *Mettl3* influences the transcriptional regulation of TEs RNAs and its broader implications for cell fate determination.

Similar to the two-cell stage (2C) of mammalian embryonic development, 2C-like cells activate the expression of MERVL and MT2 transposons, along with other totipotency-associated genes such as Zscan4, Dux, and the Usp17l families (Falco et al. 2007; Macfarlan et al. 2012; Hendrickson et al. 2017). Due to their totipotency-like properties, 2C-like cells have been shown, through chimera experiments, to contribute to both embryonic and extraembryonic tissues (Macfarlan et al. 2012; Choi et al. 2017; Genet and Torres-Padilla 2020). Although previous studies have shown METTL3 deficiency enhances 2 C gene expression (Liu et al. 2021), there is currently no direct evidence indicating that *Mettl3* KO promotes 2C-like program. Furthermore, prior research using *Mettl3* KO and wild-type (WT) mESCs in embryonic chimera experiments revealed that *Mettl3* KO ESCs exhibited impaired contributions to chimeric embryos at the E12.5 stage (Geula et al. 2015).

Here, we report that *Mettl3* KO leads to widespread upregulation of TEs expression in an m^6^A enzyme activity-dependent manner and independent of culture conditions. Additionally, 2C-like gene expression also increases in *Mettl3* KO mESCs in an m^6^A enzyme activity-dependent manner. Embryo chimeric experiments confirm that METTL3 deficiency activates 2C-like program in mESCs.

## Results

### *Mettl3* KO leads to widespread upregulation of TEs expression in a m^6^A enzyme activity-dependent manner, regardless culture conditions

Previous studies have shown that METTL3 deficiency in mESCs enhances the expression of SETDB1-YTHDC1-dependent H3K9me3-marked TEs in serum-containing (serum + LIF) culture conditions (Liu et al. 2021). However, another study (Chelmicki et al. 2021) reported that in serum-free (N2B27 + 2i + LIF) culture conditions, *Mettl3* knockout in mESCs led to upregulation of IAP mRNA levels, while LINE transcripts showed opposite downregulation.

**Fig. 1:**
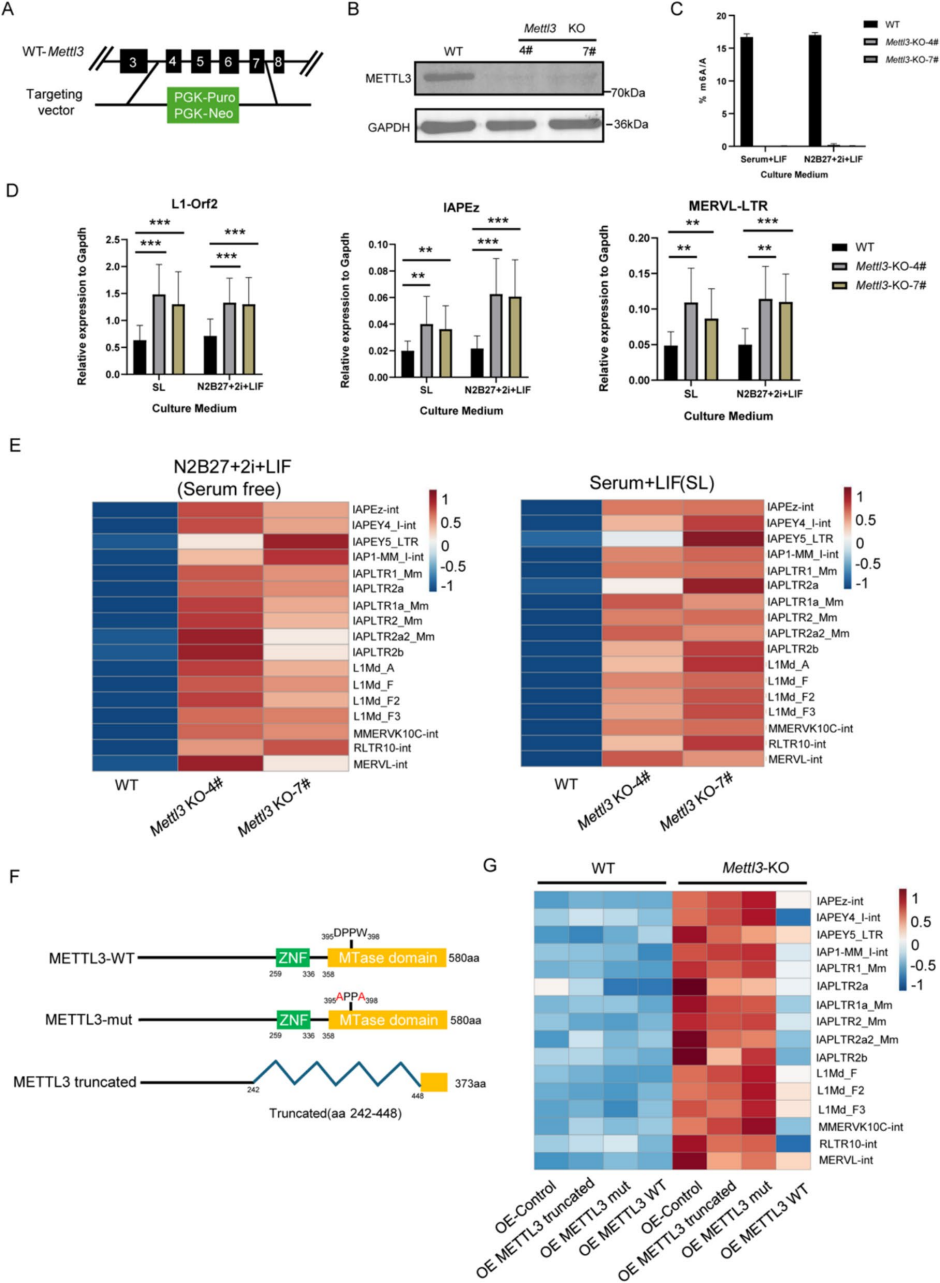
*Mettl3* KO induces TEs expression in a m^6^A enzyme activity-dependent manner, regardless culture conditions (**A**) Schematic diagram showing the strategy of *Mettl3* KO cell lines. **B** Western blot verifies the generation of *Mettl3*-KO cell lines. **C** Mass spectrometry detecting the ratio of m^6^A to A in the mRNA of WT and *Mettl3*-KO cell lines serum+LIF and serum-free (N2B27+2i+LIF) culture conditions. Data are mean ± s.d. of 2 independent experiments. *n*=2 biological replicates. **D** RT-qPCR data demonstrates the expression of TEs RNA in the WT and* Mettl3*-KO cell lines in serum+LIF and serum-free (N2B27+2i+LIF) culture conditions. *n*=2 biological replicates. Data are mean ± s.d. of 4 independent experiments. *P* values determined by two-sided Student’s t-test. **E** Left, heat map of RNA-seq data showing the expression changes of indicated TEs upon *Mettl3* depletion in serum-free (N2B27+2i+ LIF) culture conditions (*n*=2 biological replicates.). Right, heat map of RNA-seq data showing the expression changes of indicated TEs upon *Mettl3* depletion in serum+ LIF culture conditions (*n*=2 biological replicates.). **F** Schematic showing the relevant protein structure of METTL3-WT, METTL3-mut and METTL3-truncated in the Overexpression Experiment (aa, amino acids). **G** Heat map of RNA-seq data showing the expression changes of indicated TEs upon *Mettl3* depletion after overexpression of full-length METTL3, METTL3 with a catalytic site mutation (DPPW to APPA at aa 395–398), truncated METTL3 (aa 242–448), and the control vector (*n*=2 biological replicates.)

To further investigate the effects of Mettl3 on the transcriptional regulation of TEs in different culture conditions, we constructed a *Mettl3* knockout (KO) mESC line using CRISPR-Cas9 gene editing technology, targeting exons 4–7 of *Mettl3* (Fig. 1A). This strategy disrupts the catalytic active motif of METTL3, DPPW, located in exon 6 (Wang et al. 2016; Akhtar et al. 2021; Mansfield 2024), and induces a frameshift mutation that abolishes METTL3's catalytic activity.

Western blot analysis confirmed that METTL3 protein was almost undetectable in both *Mettl3* KO cell lines (Fig. 1B). We cultured WT and *Mettl3* KO mESCs in serum + LIF and N2B27 + 2i + LIF conditions. *Mettl3* KO mESCs exhibited differential growth patterns depending on the culture conditions. In serum + LIF, *Mettl3* KO mESCs displayed spindle-shaped monolayers with large intercellular spaces and were unable to form colonies. In contrast, in N2B27 + 2i + LIF, *Mettl3* KO mESCs formed flattened, colony-like structures (Fig. S1A).

qPCR analysis showed that Mettl3 transcription was almost undetectable in *Mettl3* KO cell lines, regardless of culture conditions (Fig. S1B). Quantitative mass spectrometry analysis of m^6^A levels in purified mRNA revealed that mRNA in *Mettl3* KO mESCs had almost no m^6^A modification, unaffected by culture conditions (Fig. 1C). These findings indicate that while *Mettl3* KO mESCs exhibit different morphological characteristics in serum-containing and serum-free culture conditions, the transcriptional expression of Mettl3 and its enzymatic activity in m^6^A modification are not influenced by the culture conditions.

To investigate the effects of METTL3 on TE transcriptional regulation, we cultured WT and *Mettl3* KO mESCs in serum + LIF and N2B27 + 2i + LIF conditions. qPCR data showed that TE RNAs, including LINE1 and IAP, were significantly upregulated in *Mettl3* KO cell lines, independent of culture conditions (Figs. 1D, S1C). Bulk RNA sequencing (RNA-seq) confirmed these findings (Fig. 1E). Similar results were also observed under serum + LIF + 2i (SL + 2i) culture conditions (Fig. S1D).

To validate that TE upregulation is m^6^A enzyme activity-dependent, we overexpressed METTL3-WT, METTL3-mut (DPPW → APPA mutation) (Wang et al. 2016; Akhtar et al. 2021; Mansfield 2024), and METTL3-truncated (lacking the ZNF domain and catalytic center) in WT and *Mettl3* KO mESCs (Fig. 1F). RNA-seq analysis revealed that only METTL3-WT restored TE RNA expression to WT levels, while METTL3-mut and METTL3-truncated had no significant effect (Fig. 1G). These results confirm that TE upregulation in *Mettl3* KO mESCs is m^6^A enzyme activity dependent.

Overall, our experiments demonstrate that the widespread upregulated expression of TEs RNA in *Mettl3* KO mESCs is m^6^A enzyme activity-dependent and unaffected by the culture conditions.

### *Mettl3* KO also upregulates 2C-like gene expression in an m^6^A enzyme activity-dependent manner, regardless culture conditions

**Fig. 2:**
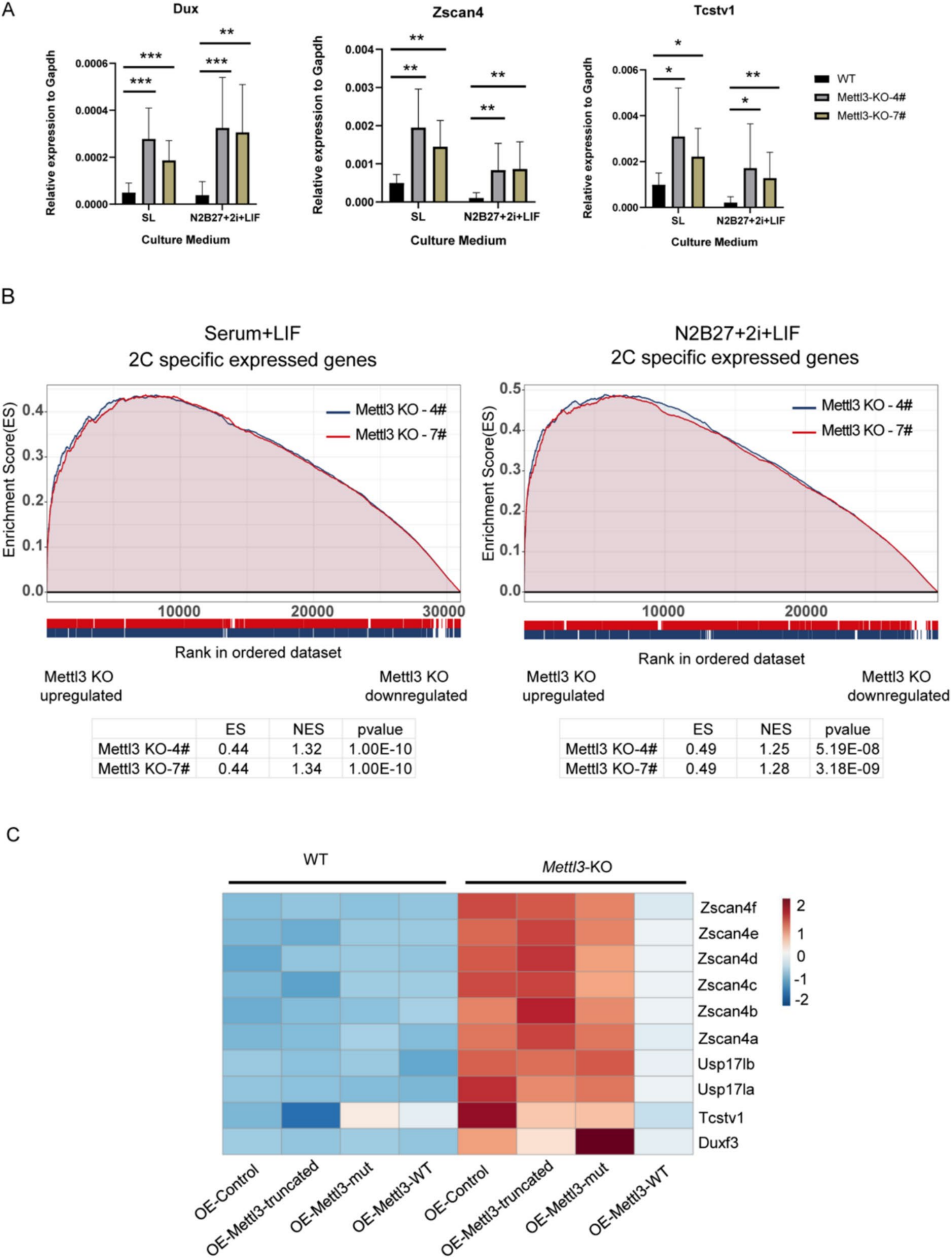
*Mettl3* KO also induces 2C-like gene expression in an m^6^A enzyme activity-dependent manner, regardless culture conditions. **A** RT-qPCR data demonstrates the expression of 2C-like genes in the WT and* Mettl3*-KO cell lines in serum+LIF and serum-free (N2B27+2i+LIF) culture conditions. Data are mean ± s.d. of 4 independent experiments. *P* values determined by two-sided Student’s t-test. *n*=2 biological replicates. **B** Left, Gene set enrichment analysis (GSEA) illustrates the upregulation of 2 C genes upon Mettl3 KO in serum+LIF culture conditions (*n*=2 biological replicates). Right, Gene set enrichment analysis (GSEA) illustrates the upregulation of 2 C genes upon *Mettl3* KO serum-free (N2B27+2i+LIF) culture conditions (*n*=2 biological replicates). **C** Heat map of RNA-seq data showing the expression changes of indicated 2C-like genes upon *Mettl3* depletion after overexpression of full-length METTL3, METTL3 with a catalytic site mutation (DPPW to APPA at aa 395–398), truncated METTL3 (aa 242–448), and the control vector (*n*=2 biological replicates)

In addition to its regulatory role in transposable elements (TEs) expression, we observed that *Mettl3* KO also led to upregulation of 2C-like gene expression, independent of culture conditions (Fig. 2A). RNA-seq data confirmed that 2C-like gene RNAs were upregulated in *Mettl3* KO mESCs in both serum + LIF and N2B27 + 2i + LIF conditions (Fig. 2B). Similar results were also observed under serum + LIF + 2i (SL + 2i) culture conditions (Fig. S2A). Overexpression of METTL3-WT, but not METTL3-mut or METTL3-truncated, restored 2C-like gene expression to WT levels (Figs. 2C, S2B). These findings confirm that the upregulation of 2C-like gene expression in *Mettl3* KO mESCs is m^6^A enzyme activity-dependent.

Overall, our results demonstrate that the upregulation of 2C-like gene expression in *Mettl3* KO mESCs is m^6^A enzyme activity-dependent and unaffected by the culture conditions.

### Embryo chimeric experiments confirm METTL3 deficiency promotes 2C-like program

**Fig. 3:**
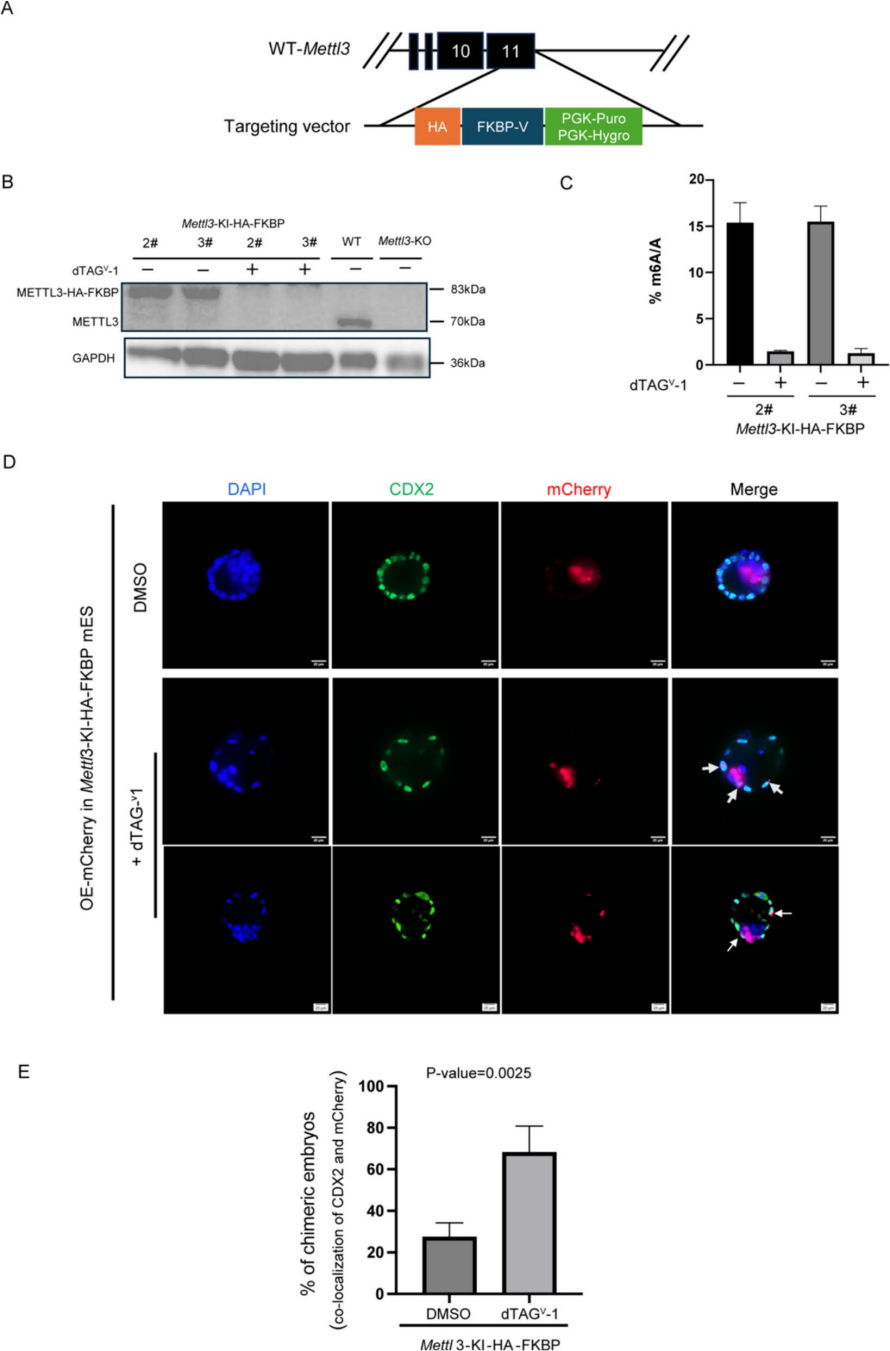
Embryo Chimeric Experiments Confirm METTL3 Deficiency Promotes Totipotency Transition. **A** Schematic diagram showing the strategy of *Mettl3*-KI-HA-FKBP cell lines. **B** Western blot verifies the generation of *Mettl3*-KI-HA-FKBP cell lines (#2 and #3) and the degradation of METTL3 protein treated with dTAG^V^−1 or not. **C** Mass spectrometry analysis of the m^6^A to A ratio in the mRNA of *Mettl3*-KI-HA-FKBP cell lines (#2 and #3) treated with dTAG^V^−1 or DMSO. *n*=2 biological replicates. **D** E3.5 blastocysts derived from the aggregation of *Mettl3*-KI-HA-FKBP mESCs (with mCherry overexpression and treated with dTAGV-1 or DMSO) and 8-cell stage embryos were stained for CDX2. Scar bars, 20 μm. *n*=2 biological replicates. **E** A column graph depicting the proportions of chimeric embryos in which *Mettl3*-KI-HA-FKBP mESCs (with mCherry overexpression and treated with dTAGV-1 or DMSO) were incorporated into trophectoderm (co-localization of CDX2 and mCherry). *P* values determined by two-sided Student’s t-test. *n*=2 biological replicates

We constructed *Mettl3*-KI-HA-FKBP mESC lines and used dTAG^V^−1 treatment to transiently degrade METTL3 protein (Fig. 3A) (Nabet et al. 2020). We then performed embryo chimeric experiments and assessed whether *Mettl3*-KI-HA-FKBP mESCs could contribute to trophectoderm lineage development in E3.5 embryos, thereby verifying whether METTL3 deficiency in mESCs expand the cellular potency.

Specifically, we knocked in FKBP-V before the stop codon of *Mettl3* in mESCs (Nabet et al. 2020). Upon treatment with 500 nM dTAG^V^−1 for 48 h (Nabet et al. 2020; Abuhashem and Hadjantonakis 2022), Western blot analysis confirmed that METTL3 protein was nearly completely degraded in the *Mettl3*-KI-HA-FKBP mESCs by adding dTAG^V^−1 (Figs. 3B, S3A). Additionally, quantitative mass spectrometry analysis of m^6^A levels in purified mRNA revealed that *Mettl3*-KI-HA-FKBP mESCs had almost no m^6^A modification after METTL3 degradation (Fig. 3C).

We then overexpressed mCherry fluorescent protein in *Mettl3*-KI-HA-FKBP mESCs (Fig. S3B) to trace whether these cells could participate in trophectoderm lineage development following METTL3 degradation. Embryo chimeric experiments demonstrated that METTL3-deficient mESCs expressing mCherry were detected in the trophectoderm lineage and colocalized with CDX2-positive cells in more than 70% of the chimeric embryos, a proportion significantly higher than that observed in the DMSO-treated control group (Figs. 3D, E). These findings suggest that METTL3 deficient mESCs contribute to trophectoderm lineage development, indicating that MEETL3 deficiency promotes the 2C-like program transition in mESCs.

## Discussion

Our study highlights the critical role of METTL3-mediated m^6^A modifications in regulating TEs and the transition of mouse embryonic stem cells (mESCs) to a 2C-like state. The high conservation of the METTL3 protein, especially its catalytic active site (Wang et al. 2016; Akhtar et al. 2021; Mansfield 2024), emphasizes its significance in m^6^A modification and subsequent biological processes.

TE-derived RNAs are enriched with m^6^A modifications (Liu et al. 2020) and METTL3 deficiency has been shown to have variable effects on different TE transcripts in different studies (Liu et al. 2021; Chelmicki et al. 2021; Xu et al. 2021). Due to the inconsistency between these research results, we initially hypothesized whether the serum components in the culture medium had different impacts on the experimental outcomes. However, after culturing wild-type (WT) and *Mettl3* knockout (KO) mouse embryonic stem cells (mESCs) under different culture conditions respectively, including serum-containing (serum + LIF) culture conditions (Liu et al. 2021) and serum-free (N2B27 + 2i + LIF) culture conditions (Chelmicki et al. 2021), it was still found that the widespread upregulated expression of transposable elements (TEs) RNA in *Mettl3* KO mESCs was not affected by the culture conditions. Similar results were also observed under serum + LIF + 2i (SL + 2i) culture conditions. Therefore, we consider that discrepancies across studies may stem from variations in knockout strategies or intrinsic heterogeneity among mESC lines.

Regarding how m⁶A deficiency leads to TE activation, multiple mechanisms have been proposed. On one hand, several studies have demonstrated that m⁶A modification promotes the degradation of TE-derived RNAs (Liu et al. 2020; Wu et al. 2022; Chelmicki et al. 2021). On the other hand, a growing number of studies suggest that m⁶A reader proteins, particularly YTHDC1, can bind to m⁶A-modified TE RNAs (e.g., LINE1, IAP, ERVK) and recruit chromatin-modifying enzymes such as SETDB1. This process reinforces H3K9me3-mediated transcriptional silencing of TEs. In this context, the loss of m⁶A results in reduced H3K9me3 deposition at TE loci, thereby facilitating their transcriptional activation (Xu et al. 2021; Liu et al. 2021; Chen et al. 2021).

To distinguish between these possibilities, we performed DRB-TT-seq (Gregersen et al. 2020) to assess newly synthesized RNA following *Mettl3* knockout. Our data revealed a marked increase in the nascent transcripts of representative TE RNAs (e.g., IAPEz-int, L1Md_Gf), indicating that their upregulation is driven primarily by transcriptional activation (Fig. S3C).

In our study, another key finding is that *Mettl3* KO upregulates 2C-like gene expression in an m^6^A enzyme activity-dependent manner. Although previous studies have shown some effects of METTL3 on 2C-like gene expression, our study further confirms the role of METTL3 deficiency in inducing 2C-like program. However, the mechanisms underlying this transition and the exact implications for embryonic development require further investigation.

While TE activation is frequently associated with the induction of the 2C-like transcriptional program (Li et al. 2024), some evidence suggests that this relationship may not be strictly causal. For example, DUX, a transcription factor specifically expressed in two-cell stage, can activates hundreds of 2C-related genes, including Zscan4 and MERVL. Moreover, ectopic expression of DUX alone is sufficient to trigger 2C-like conversion in mESCs (Hendrickson et al. 2017). However, in our previous study, we observed that in the *Dux* knockout background, *Ythdc1* knockout leads to the activation of TE families (e.g., LINE1, IAP, ERVK) but does not activate 2C-related genes (Liu et al. 2021), suggesting that the upregulation of TE subtypes (e.g., LINE1, IAP, ERVK) alone is not sufficient to induce the 2C-like program. Nevertheless, recent studies have demonstrated that endogenous activation of MERVL, a TE specifically associated with the 2 C stage, is sufficient to drive 2C-like cells with robust activation of 2 C genes and expanded potency (Yang et al. 2020). Furthermore, depletion of MERVL transcripts causes embryonic lethality due to defects in early lineage specification and genome stability, highlighting its essential role in preimplantation development (Sakashita et al. 2023). Taken together, these findings suggest that while certain TEs (e.g., LINE1, IAP, ERVK) have no direct association with the 2 C state, MERVL plays a direct and indispensable role in regulating the 2 C program.

Overall, our study has opened up new avenues for research into the role of METTL3 and m^6^A modifications in early embryonic development and cell fate determination. Future studies should focus on elucidating the molecular mechanisms by which METTL3 regulates TE transcription and 2C-like program induction, as well as its implications for developmental abnormalities related to epigenetic dysregulation.

## Conclusions

In summary, our study reveals that loss of METTL3-mediated m⁶A modifications in mESCs leads to widespread upregulation of transposable elements (TEs) and 2C-like genes in an m^6^A enzyme activity-dependent manner, regardless of culture conditions. Moreover, METTL3 deficient mESCs contribute to trophectoderm lineage development, indicating that MEETL3 deficiency promotes the 2C-like program transition in mESCs. Together, this study emphasize that METTL3-dependent m⁶A methylation plays a key role in regulating both gene expression and developmental plasticity in mESCs and further implicate m⁶A loss in promoting a 2C-like program associated with enhanced developmental potential.

## Materials and methods

### Cells culture and maintenance

Male mouse ES cells were derived from 3.5 days post coitum dpc inner cell mass (ICM) from 129 mice (stock no.217, Beijing Vital River Laboratory Animal Technology). Genetically engineered mouse ES cells, including *Mettl3* KO, *Mettl3*-KI-HA-FKBP and OE-mCherry in *Mettl3*-FKBP mES cells were constructed and cultured in feeder-free conditions and feeder-dependent conditions.

For feeder-free culture condition, mES and *Mettl3* KO cells were grown in three different media:

1. Serum + LIF (SL) medium, which is ccomposed of high-glucose Dulbecco’s modified Eagle’s medium (HG DMEM, HyClone, SH30022.01), 15% fetal bovine serum (FBS, Lonsera, s711-001 s), 1 × GlutaMAX (GLUTAMAX, gibco, 35050079), 1 × nonessential amino acids (NEAA, Gibco, 11140076), 1 × sodium pyruvate (Gibco, 11360070), 1 mM 2-mercaptoethanol (Gibco, 21985023) and 1000 U/mL LIF (LIF, Novoprotein, c690).
2. N2B27 + 2i + LIF medium which consists of 50% HG DMEM (Hyclone), 50% KNOCKOUT™ D-MEM (Gibco, 10829018), 1 × GlutaMAX (GLUTAMAX, gibco, 35050079), 1 × nonessential amino acids (NEAA, Gibco, 11140076), 1 mM 2-mercaptoethanol (Gibco, 21985023), N2 (gibco, 17502048), B27 (Gibco, 7504044), 3 µM GSK3 inhibitor (CHIR99021, DC, DC1023), 1 μM MEK inhibitor (PD0325901, DC, DC1056) and 1000 U/mL LIF (LIF, Novoprotein, c690).
3. serum + LIF + 2i (SL + 2i) medium, which is ccomposed of high-glucose Dulbecco’s modified Eagle’s medium (HG DMEM, HyClone, SH30022.01), 15% fetal bovine serum (FBS, Lonsera, s711-001 s), 1 × GlutaMAX (GLUTAMAX, gibco, 35050079), 1 × nonessential amino acids (NEAA, Gibco, 11140076), 1 × sodium pyruvate (Gibco, 11360070), 1 mM 2-mercaptoethanol (Gibco, 21985023), 3 µM GSK3 inhibitor (CHIR99021, DC, DC1023), 1 μM MEK inhibitor (PD0325901, DC, DC1056) and 1000 U/mL LIF (LIF, Novoprotein, c690).

For feeder-dependent culture, *Mettl3*-KI-HA-FKBP and OE-mCherry in *Mettl3*-FKBP mES cells were culture on growth-arrested moused embryonic fibroblasts (MEFs), according to a modified protocol derived from WISC Bank. Briefly, MEFs treated with mitomycin (Stem cell, 73,274) were plated on 0.2% gelatin-coated (w/v, homemade) six-well plates at a density of 4.0 × 10^5^ cells per well one day before splitting or thawing cells. The feeder dependent cells were maintained in the SL + 2i medium. All cell lines were cultured at 37 °C in a humidified atmosphere containing 5% CO2.

dTAG^V^−1 (Bio-Techne) was reconstituted in DMSO (Sigma) at a concentration of 1 mM and diluted in maintenance medium to 500 nM before being added to cells, with medium changes occurring at specified time intervals. Mycoplasma detection tests were conducted routinely to ensure mycoplasma-free conditions throughout the study.

### Generation of gene-editing cell lines

For generation of *Mettl3*-KO mouse ES cells, 129 mouse ES cells were targeted for disruption of the endogenous *Mettl3* locus using the CRISPR–Cas9 system and homologous recombination. Exons 4–7 of mouse Mettl3 were deleted on the basis that they contain the DPPW active motif. Next, the gRNAs and the linearized targeting vector were electroporated into the mouse ES cells using the Mouse Embryonic Stem Cell Nucleofector Kit (Lonza, VPH-1001). After screening (1 μg/mL puromycin, 400 μg/mL G418 or 200 μg/mL hygromycin) for 3–5 days, colonies were picked and validated by genotyping PCR or western blot.

For generation of *Mettl3*-KI-HA-FKBP mouse ES cells, two guide RNAs (gRNAs) were strategically designed to target the 3’-untranslated region (3-UTR), approximately 200 bp downstream from the stop codon. The sequences of guide RNAs used in this study are shown in Supplementary Table 1. To assemble the Cas9/sgRNA RNPs, we incubated purified Cas9 protein (Novoprotein, E365-01A) with individual gRNA at room temperature for 10 min and then mix them together. The mixture and the linearized targeting donor plasmid DNA were electroporated into the mES cell line using the Mouse Embryonic Stem Cell Nucleofector Kit (Lonza, VPH1001). After resistance screening (1 μg/mL puromycin and 200 μg/mL hygromycin) for 3–5 days, surviving colonies were isolated and subjected to validation using long-range PCR and second-generation sequencing to confirm the precise integration of the FKBP tag at the desired locus within the *Mettl3* gene.

### Plasmid construction and cell transfection

The coding sequences for mouse *Mettl3*, *Mettl3*-mut (APPA), and *Mettl3*-truncated transcripts were amplified from mouse embryonic stem (mES) cell cDNA and subsequently cloned into a modified pB-CAG vector. This vector features a P2A tag and a PGK-puromycin or PGK-hygromycin selection marker, which are positioned upstream of the *BamHI* restriction enzyme site. The vector was digested with EcoRI and MluI to facilitate the cloning of the *Mettl3*, *Mettl3*-mut (APPA), and *Mettl3*-truncated coding sequences using the same restriction enzymes with the following primers: forward primer 5’-CATCATTTTGGCAAAGAATTCatgtcggacacgtggagctc-3’ and reverse primer 5’-AGCAGGCTGAAGTTAGTAGCGGGGGGCGACGCGTTTActataaattcttaggtttagagatgatgccgt-3’. WT and *Mettl3* KO ES cells were plated onto 12-well dishes at a density of 2 × 10^5^ cells per well. According to the manufacturer's instructions, Lipofectamine™ 3000 (Invitrogen, USA) was used to transfect distinct groups of ES cells with *Mettl3*、*Mettl3*-mut(APPA) and *Mettl3*-truncated plasmid. The control cell line with expression of EGFP was created similarly. After transfection, the culture medium was changed every 8 h, and subsequent overexpression of related proteins was performed 48 h after transfection. After four days, the cells were treated with 0.25% trypsin to detach them from the culture plates and then collected for reverse transcription quantitative polymerase chain reaction (RT-qPCR) analysis.

### Western blot

Western blots were performed using typical laboratory procedures with the following antibodies: anti-METTL3 (Proteintech, 150731–1-AP) and anti-GAPDH (MAB374, Millipore). Briefly, cells were lysed on ice in SDS buffer (62.5 mM Tris–HCl (pH 6.8 at 25℃)). Whole-cell extracts were resolved by 10% or 12% SDS-PAGE, transferred to PVDF membranes and probed with corresponding antibodies according to the manufacturer’s recommendations (Cell Signaling Technology). Antibodies used in this study are listed in the Supplementary Table 2.

### RNA extraction and RT–qPCR analysis

To extract the total RNAs, cells were collected and lysed with 1 mL TRIzol reagent (MRC, TR118-200), followed by addition of 200 μl chloroform. After centrifugation (14,000 g for 10 min at 4 °C), the total RNAs in the supernatant were precipitated with isopropanol. RNA quantity and quality were assessed with NanoDrop One C spectrophotometer (ND-ONEC-W, Thermo Fisher Scientific). Only RNA with an absorbance read ratio 260/280 between 1.8 and 2.0 was used for exper iments. Then, 1 µg of total RNA was used for cDNA synthesis with HiScript II Q RT SuperMix for qPCR (R22201, Vazyme), and the diluted cDNA was used as template for qPCR with ChamQ SYBR qPCR Master Mix (Q311-02, Vazyme). Furthermore, real-time quantitative PCR analysis was performed on the CFX96 real-time system (Bio-Rad) using ChamQ Universal SYBR qPCR master mix (Vazyme, Q711-02). Gene expression levels were normalized to those of Gapdh. qPCR primers used in this study are shown in Supplementary Table 3. All reactions were run in QuantStudio 3 Real-Time PCR System (Thermo Fisher Scientific).

### LC–MS/MS for detection and quantification of RNA modifications

LC–MS/MS was performed as previously described (Zhang et al. 2021). In brief, 200 ng extracted RNA from mESCs was digested into nucleosides by Nuclease P1 (1 U, NEB, M0660S) and shrimp alkaline phosphatase (rSAP, 1 U, NEB, M0371S) in 50 μl RNase-free water at 37 °C overnight. The mixture was diluted to 100 μl, 10 μl of which was injected into an LC–MS/MS system consisting of a high-performance liquid chromatographer (Shimadzu) equipped with a C18-T column (Weltech) and a Triple Quad 4500 (AB SCIEX) mass spectrometer in positive ion mode by multiple-reaction monitoring. Mass transitions of *m*/*z* 268.0–136.0 (A), *m*/*z* 245.0–113.1 (U), *m*/*z* 244.0–112.1 (C), *m*/*z* 284.0–152.0 (G) and* m*/*z* 282.0–150.1 (m^6^A) were monitored and recorded. A concentration series of pure commercial nucleosides (MCE) was employed to generate standard curves. Concentrations of nucleosides in samples were obtained by fitting signal intensities to standard curves with certain ratios calculated subsequently.

### RNA-seq and data analysis

Total RNAs were extracted as described above. The Total RNA-seq (H/M/R) Library Prep Kit for Illumina (Vazyme, NR605) was used for RNA library preparation. In brief, 1 μg total RNAs were hybridized with the rRNA probe (H/M/R) and digested by RNase H to remove ribosomal RNAs. After DNase I digestion, the ribosomal-depleted RNAs were fragmented at 94 °C for 8 min. Then, the first-strand and second-strand cDNAs were synthesized successively using the provided reagents. The cDNA was purified by VAHTS DNA Clean Beads (VAHTS, NR411), followed by adaptor ligation. After purification, the cDNAs were PCR-amplified and purified using VAHTS DNA Clean Beads. The RNA-seq data processing was performed as described (He et al. 2019). For TE-expression analysis, the reads were mapped to the mouse genome mm10 using the STAR aligner (Dobin et al. 2013) and the counts for each TE were calculated using the scTE (He et al. 2019). Normalization was performed by measuring the counts per million (cpm) at all TEs.

### Genotype

The rapid DNA extraction was accomplished using Viagen DirectPCR lysis buffer, enhanced with the addition of 20 mg/mL fresh proteinase K to each well containing adhesive living cells. The solution in the wells was pipetted up and down several times using a multichannel pipet and was ultimately transferred into 200 ul PCR tubes. These tubes were incubated at 56 °C for 2 h to lyse the cells, followed by a heat treatment at 85 °C for 10 min to inactivate the proteinase K. Afterward, the tubes were loaded into a centrifuge to spin down the debris at 250 g for 1–2 min. Supernatants containing the extracted DNA were further purified by precipitation. The amplifications were performed at 94 °C for 3 min, followed by 35 cycles of denaturation at 94 °C for 30 s, 56 °C for 30 s and elongation at 72 °C for 1 min, 72 °C for 5 min. PCR product was electrophoresed on 1% agarose gel and visualized by Bio-Rad gel imager.

### Chimeric blastocyst assay

The zona pellucida of the 8-cell stage embryos were removed by a short exposure to acidic Tyrode’s solution (Sigma, T1788). Two denuded embryos were placed into each of the concaved microwells created by an aggregation needle. Ten to fifteen OE-mCherry in *Mettl3*-FKBP mES cells were transferred into each concaved microwell and cocultured with the denuded embryos. Before and after the aggregation procedure, all embryos were cultured in potassium simplex optimized medium (KSOM) medium^4^. To obtain E3.5 chimeric embryos for observing contribution of ES cells and immunostaining, the aggregated embryos were cultured at 37 °C in 5% CO2 for 24 h.

### Immunofluorescence staining

Whole-mount immunofluorescence staining of E3.5 embryos was performed as previously described (Wu et al. 2013). In brief, embryos were fixed in 4% paraformaldehyde at room temperature for 30 min, washed 3 times in PBS-T (PBS + 0.1% Triton X-100), and permeabilized for 0.5 h in PBS 0.3% Triton X-100. E3.5 embryos were blocked in PBS-T with 1% BSA (Sigma) and 3% normal donkey serum (Solarbio) at room temperature for 2 h. Primary and secondary antibodies were diluted in the blocking solution. E3.5 embryos were incubated with primary antibodies at 4 °C overnight. The following primary antibody called mouse anti-CDX2 (1:200; BioGenex, MU392A-UC) was used. After three washes in PBS-T, the embryos were incubated with secondary antibodies. Finally, the embryos were washed 4 times in PBS-T and stained in 10 μg/mL DAPI for 1 h and mounted in PBS in a glass-bottom dish for observation. The confocal microscope (Olympus, IXplore SpinSR) was operated with a 60 × oil lens with 405, 488 and 647 nm lasers.

### DRB-TT-seq

DRB-TT-seq was performed as previously described (Gregersen et al. 2020), with minor modifications. In brief, *Mettl3*-KO mouse ES cells and 129 mouse ES cells at ~ 80% confluency were pulse-labeled with 1 mM 4-thiouridine (4sU) (Sigma, T4509) for 0 and 8 min. To capture newly transcribed RNA, these cells were pre-treated with 100 μM DRB (Sigma, D1916) for 3 h to inhibit RNA Pol II transcription. After DRB removal by washing three times with PBS, transcription was reinitiated, and newly transcribed RNA was labeled with 1 mM 4sU for 0 and 8 min. Cells were lysed in TRIzol and stored at −80 °C. Total RNA was extracted using chloroform and isopropanol precipitation with glycogen/DTT. 100–150 µg total RNA (including 5 µg S2 spike-in RNA) was fragmented in 0.2 M NaOH on ice for 14 min. The reaction was neutralized with 1 M Tris–HCl (pH 6.8). Fragmented RNA was biotinylated with MTSEA-biotin-XX (Biotium, #90066) at a final concentration of 0.167 mg/mL for 30 min to 30 min-2 h at room temperature in a buffer containing 10 mM HEPES–KOH (pH 7.5) and 1 mM EDTA. Unreacted biotinylation reagent was removed by chloroform extraction followed by purification using RNA Clean XP Beads and isopropanol precipitation. Dynabeads MyOne Streptavidin C1 beads (Invitrogen, 65001) were blocked with yeast tRNA (200 ng/µL) at room temperature for 1 h. Biotinylated RNA was captured by 45 µl C1 beads at room temperature for 15 min. Beads were washed 5 times with 1 ml binding and wash buffer (100 mM Tris–HCl pH 7.5, 1 M NaCl, 10 mM EDTA, 0.05% (v/v) Tween 20 in DEPC-Treated water), and eluted with 100 mM DTT in binding and wash buffer. The eluted nascent RNA was purified using RNA Clean XP Beads and isopropanol precipitation. 50–100 ng purified nascent RNA was used for library construction starting from first-strand synthesis, using a commercial kit (Vazyme, NR605) following the manufacturer’s instructions, without rRNA depletion or size selection.

### Statistical analysis

Data were performed in triplicates and presented as mean ± SD or mean ± SEM. *P*-values were determined by two-tailed Student’s test (t-test), and statistics were *P* value < 0.05 were considered statistically signifcant. *p < 0.05; **, *p* < 0.01; ***, *p* < 0.001.

## Supplementary Information

Additional file 1: Supplementary Figures. Fig S1, Fig S2, Fig S3.

Additional file 2: Supplementary Table 1. guide RNA sequence.

Additional file 3: Supplementary Table 2. antibody information.

Additional file 4: Supplementary Table 3. qPCR primer for RT-qPCR.

## Data Availability

The data supporting the findings of this study are available from the corresponding author upon reasonable request. The datasets generated and analyzed during the current study have been deposited in the BioProject database under accession number PRJCA040908.

## Abbreviations

METTL3: Methyltransferase-like 3
KO: Knockout
mESCs: Mouse embryonic stem cells
TEs: Transposable elements
m^6^A: N6-methyladenosine
LINEs: Long Interspersed Nuclear Element-1
IAPs: Intracisternal A-particles
ERVs: Endogenous retroviral elements
2C: 2 Cell
WT: Wildtype
SINE: Short Interspersed Nuclear Element
MERVL: Murine Endogenous RetroVirus-L
Zscan4: Zinc Finger and SCAN Domain Containing 4
DUX: Double Homeobox
Tcstv1: Two-cell stage variable transcript 1
Tcstv3: Two-cell stage variable transcript 3
IAPEz: Intracisternal A-particle Endogenous Retrovirus epsilon
OE: Overexpression
