# Supplementary material for: The role of METTL3 in transposable elements regulation and 2C-like program induction in mouse embryonic stem cell
Source: Cell Regen. 2025 Nov 20;14:47. doi: 10.1186/s13619-025-00262-w (PMC12635009; doi:10.1186/s13619-025-00262-w)
Supplement: Supplementary file 1 — Additional file 1: Supplementary Figures. Fig S1, Fig S2, Fig S3. [file 13619_2025_262_MOESM1_ESM.docx]

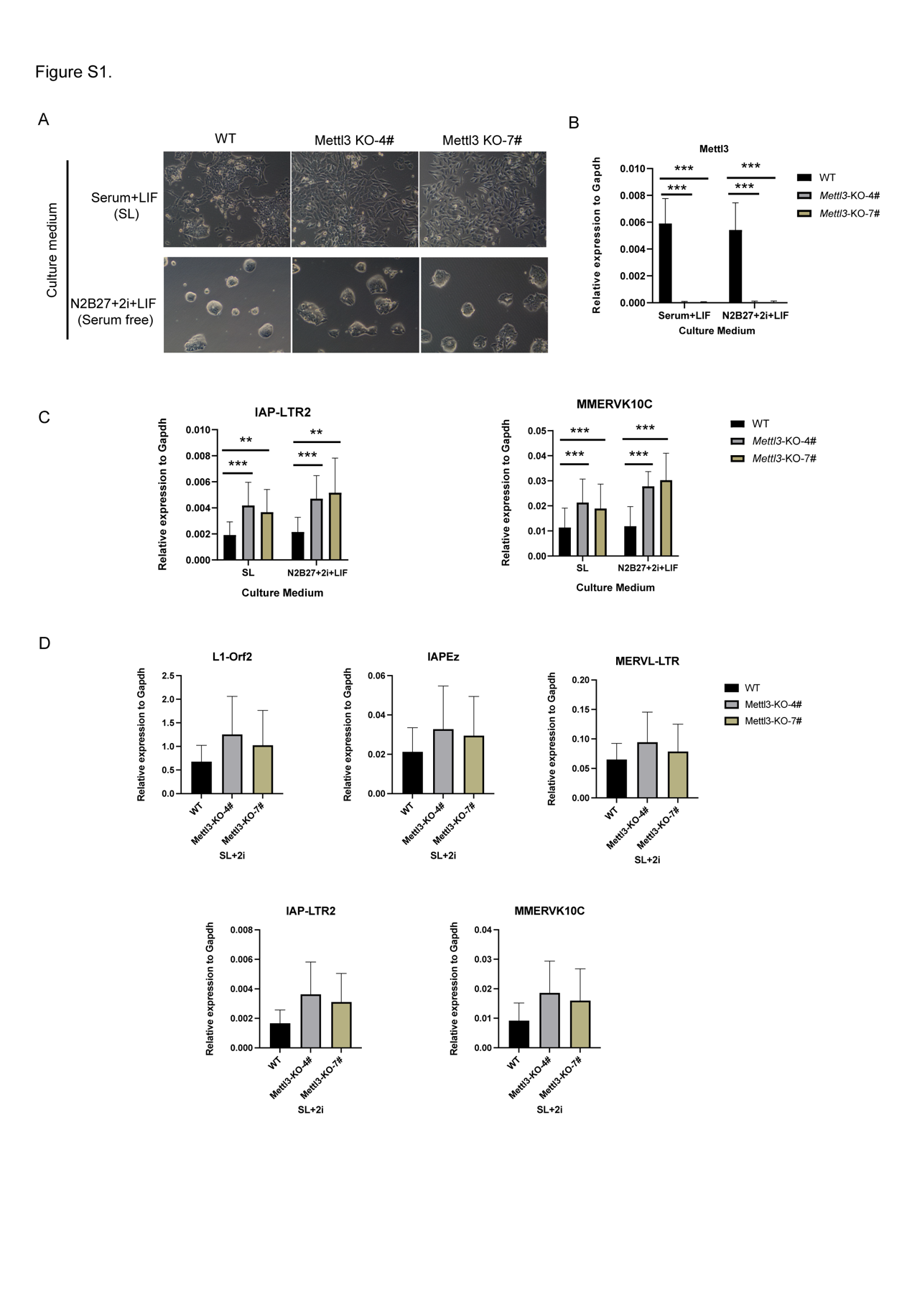


**Supplementary Fig. S1** (**A**) Phase-contrast morphological characteristics display the WT and *Mettl3* KO cell lines in serum-containing（serum+LIF） and serum-free（N2B27+2i+LIF） culture conditions. Images are representative of at least two independent experiments. (**B-C**) RT-qPCR data demonstrates the expression of Mettl3 and the expression of TEs RNA in the WT and *Mettl3*-KO cell lines in serum+LIF and serum-free (N2B27+2i+LIF) culture conditions. Data are mean ± s.d. of 4 independent experiments. P values determined by two-sided Student’s t-test. n=2 biological replicates. (D) RT-qPCR data demonstrates the expression of TEs RNA in the WT and *Mettl3*-KO cell lines in serum+LIF+2i (SL+2i) culture conditions. Data are mean ± s.d. of 4 independent experiments. n=2 biological replicates.


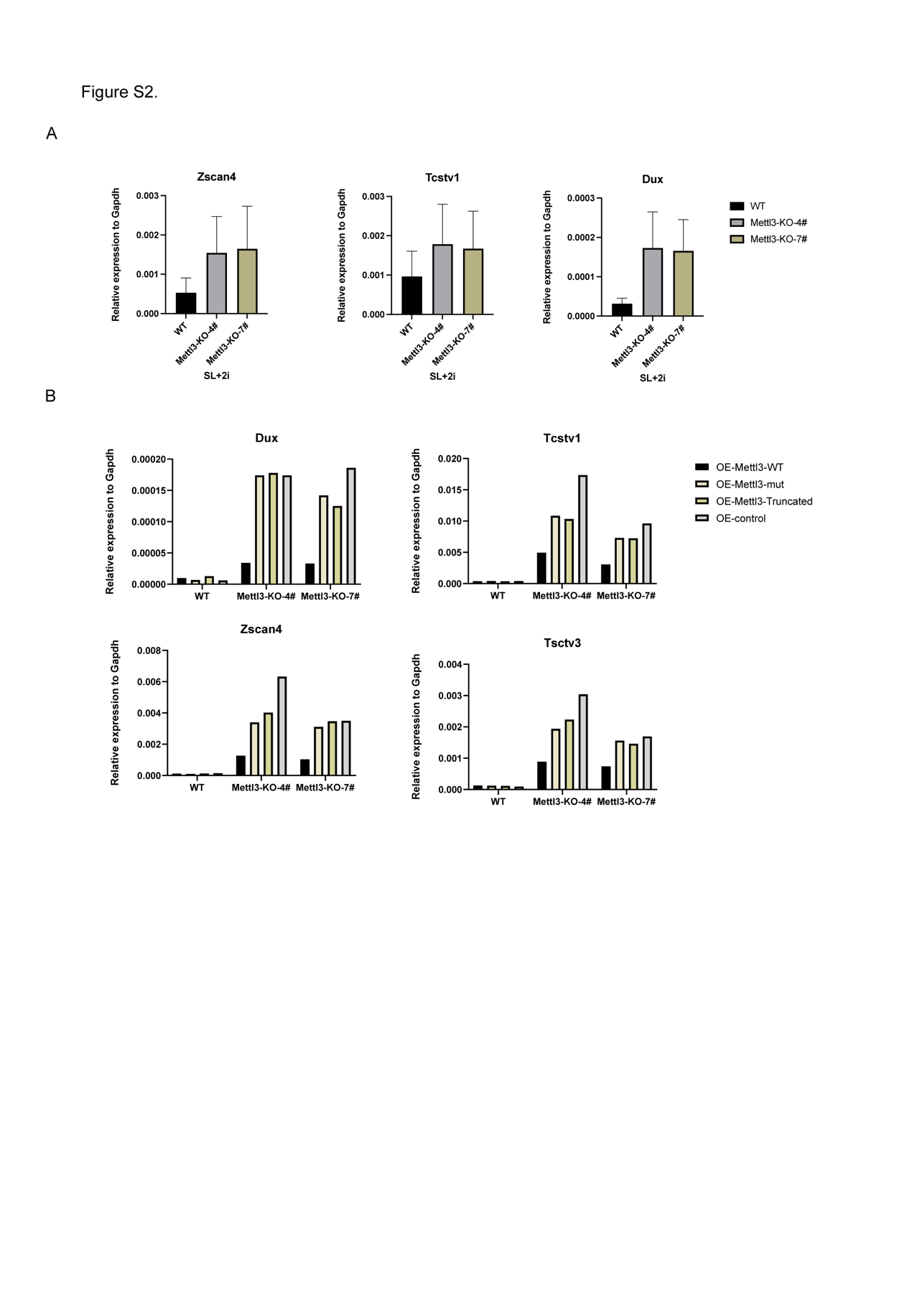


**Supplementary Fig. S2** (**A**) RT-qPCR data demonstrates the expression of 2C-like genes in the WT and *Mettl3*-KO cell lines in serum+LIF+2i (SL+2i) culture conditions. Data are mean ± s.d. of 4 independent experiments. n=2 biological replicates. (**B**) RT-qPCR data demonstrates the expression changes of indicated 2C-like genes upon *Mettl3* KO after overexpression of full-length METTL3, METTL3 with a catalytic site mutation (DPPW to APPA at aa 395-398), truncated METTL3 (aa 242-448), and the control vector. n=2 biological replicates.


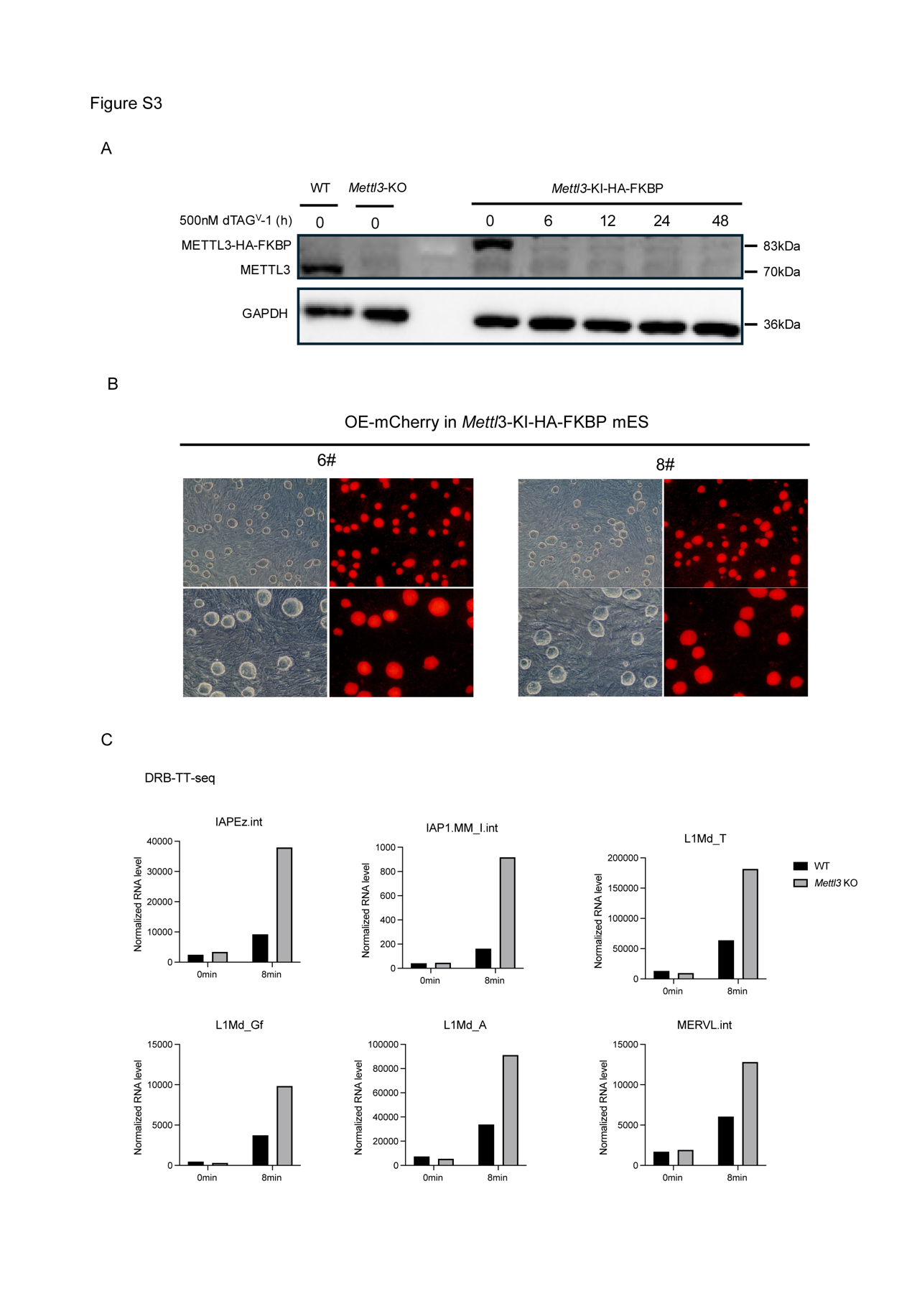


**Supplementary Fig. S3 (A)** Time-course analysis of METTL3 degradation in *Mettl3*-KI-HA-FKBP mESCs treated with 500 nM dTAG^V^-1. western blot showing METTL3 protein levels at 0, 6, 12, 24, and 48 hours after dTAG^V^-1 treatment. (**B**) Phase-contrast and fluorescence images show the *Mettl3*-KI-HA-FKBP mESCs. Images are representative of at least two independent experiments. n=2 biological replicates. (**C**) Analysis of newly transcribed TEs RNA at the indicated time points after DRB removal.
